# Pertussis surveillance results from a French general practitioner network, France, 2017 to 2020

**DOI:** 10.2807/1560-7917.ES.2022.27.17.2100515

**Published:** 2022-04-28

**Authors:** Marion Debin, Titouan Launay, Louise Rossignol, Fatima Ait El Belghiti, Sylvain Brisse, Sophie Guillot, Nicole Guiso, Daniel Levy-Bruhl, Lore Merdrignac, Julie Toubiana, Thierry Blanchon, Thomas Hanslik

**Affiliations:** 1 Sorbonne Université, INSERM, Institut Pierre-Louis d’Épidémiologie et de santé publique, Paris, France; 2 Université Paris Cité, Département de médecine générale, Paris, France; 3 Santé publique France, Département des maladies infectieuses, Saint-Maurice, France; 4 Institut Pasteur, National Reference Center for Whooping Cough and other Bordetella Infections, Paris, France; 5 Institut Pasteur, Université Paris Cité, Biodiversity and Epidemiology of Bacterial Pathogens Unit, Paris, France; 6 Institut Pasteur, Paris, France; 7 Epiconcept, Paris, France; 8 Université Paris Cité, Service de Pédiatrie Générale et Maladies Infectieuses, Hôpital Necker -Enfants malades, Assistance Publique-Hôpitaux de Paris, AP-HP, Paris, France; 9 Université Versailles-Saint-Quentin-en-Yvelines, UVSQ, UFR des sciences de la santé Simone-Veil, Versailles, France; 10 Assistance Publique-Hôpitaux de Paris, AP-HP, Hôpital Ambroise Paré, Service de Médecine Interne, Boulogne Billancourt, Paris, France

**Keywords:** whooping cough, Bordetella, sentinel network, surveillance, pertussis vaccine, France

## Abstract

**Introduction:**

In France, three complementary surveillance networks involving hospitals and paediatrician practices currently allow pertussis surveillance among infants (<1 year old) and children (1–12 years old). Data on incidences among adolescents (13–17 years old) and adults (≥ 18 years) are scarce. In 2017, a sentinel surveillance system called Sentinelles network, was implemented among general practitioners (GPs).

**Aim:**

The purpose of Sentinelles network is to assess pertussis incidence, monitor the cases’ age distribution and evaluate the impact of the country’s vaccination policy. We present the results from the first 4 years of this surveillance.

**Methods:**

GPs of the French Sentinelles network reported weekly numbers of epidemiologically or laboratory-confirmed cases and their characteristics.

**Results:**

A total of 132 cases were reported over 2017–2020. Estimated national incidence rates per 100,000 inhabitants were 17 (95% confidence interval (CI): 12–22) in 2017, 10 (95% CI: 6–14) in 2018, 15 (95% CI: 10–20) in 2019 and three (95% CI: 1–5) in 2020. The incidence rate was significantly lower in 2020 than in 2017–2019. Women were significantly more affected than men (83/132; 63% of women, p = 0.004); 66% (87/132) of cases were aged 15 years or over (median age: 31.5 years; range: 2 months–87 years). Among 37 vaccinated cases with data, 33 had received the recommended number of doses for their age.

**Conclusions:**

These results concur with incidences reported in other European countries, and with studies showing that the incidences of several respiratory diseases decreased in 2020 during the COVID-19 pandemic. The results also suggest a shift of morbidity towards older age groups, and a rapid waning of immunity after vaccination, justifying to continue this surveillance.

## Introduction

Pertussis, commonly known as whooping cough, is a highly contagious respiratory infection, mainly due to *Bordetella pertussis*. It typically results in potentially severe disease in infants (<1 year old) and young children (1–5 years old) and in a mild but prolonged cough in adults (≥18 years). Adults or adolescents (13–17 years old) have been shown to generally be the source of infection of infants hospitalised with pertussis [1,2]. Despite an initial decline of incidence following the introduction of mass vaccination of children (1–12 years old) in the mid-20^th^ century, pertussis continues to impose an important burden worldwide, and remains a major public health concern [3].

In France, pertussis was a notifiable disease between 1947 and 1986. In 1950, more than 5,000 cases were notified, including 604 deaths. After the introduction of pertussis vaccination in 1959, the annual incidence decreased massively (in 1985, 86 cases were notified, including one death) [4]. This likely reflected a low circulation of the bacterium and an underdiagnosis/notification of cases, which led health authorities to stop the mandatory surveillance in 1986 [4,5].

Since 1996, pertussis surveillance is conducted among infants by the ‘Réseau national de la coqueluche’ (Renacoq), a hospital network including more than 40 participating hospitals [1]. Between 1996 and March 2016, children and young adolescents (aged 13–16 years) were also included in this surveillance. Microbiologists involved in the network currently report pertussis culture and PCR laboratory results for hospitalised infants, while paediatricians declare and describe clinical, biological and epidemiological characteristics of hospitalised infant cases less than 6 months of age. The reporting/declarations go to Santé publique France (the French National Public Health Agency), which monitors Renacoq. Microbiologists are encouraged to send the isolates to the National Reference Center for Whooping Cough, and typically do so [6]. In 2015, the European Centre for Disease Prevention and Control (ECDC) set up ‘Pertussis in Infants European Network’ (PERTINENT), a hospital-based active pilot surveillance system that involves 41 hospitals from six countries, including 21 French hospitals participating in the Renacoq surveillance [7]. This system monitors laboratory-confirmed cases of pertussis (by PCR and/or culture) among infants attending hospitals participating in the network [7].

Since 2002, a French paediatric ambulatory surveillance system (including around 60 paediatricians in private practices) monitors in France the duration of immunity conferred by pertussis-containing vaccines and identifies changes in epidemiology of pertussis [8].

As pertussis is no longer a notifiable disease in France, data on incidences among adolescents and adults are scarce. The last study conducted in this population was carried out in 2013–2014 in general practice among people aged ≥ 50 years old, showing an incidence of 103.6 cases per 100,000 inhabitants [9].

The recommended immunisation schedule for pertussis in France has changed several times over the last 20 years. The current schedule is described in the Box below. Whole-cell vaccines were progressively replaced by acellular vaccines, and are no longer available in France since 2005. The immunisation coverage in 21-month-old children (primary immunisation + first booster) has increased from 84.1% for children born in 2017 to 90.5% for children born in 2019 [10]. Among adults, the fourth booster coverage is still low, despite an increase from 22% in 2009 to 61% in 2014 for mothers, and from 21% in 2010 to 42% in 2013 for fathers, owing to a cocooning strategy [11]. In 2017, the booster coverage was 36.0% among 29 year-olds, 25.0% among 49 year-olds, 21.7% among 69 year-olds [12].

Box **Summary of the current recommended immunisation schedule for pertussis in France [34], 2022**

For everybody:

- A primary immunisation at 2 and 4 months of age (mandatory for all children born after 1 January 2018)

- A booster at 11 months of age (mandatory for all children born after 1 January 2018)

- A second booster at 6 years of age

- A third booster between 11 and 13 years of age

- A fourth booster recommended for adults at 25 years of age with a catch-up booster proposed up to 40 years of age

For specific populations:

- A booster proposed for people in contact with infants below 6 months of age (the ‘cocooning strategy’)

- A booster recommended at 25, 45 and 65 years of age for health professionals and childcare professionals

- A booster for pregnant women, during each pregnancy (optimally between 20 and 36 weeks of amenorrhoea) [37]

Given the lack of data in primary care (in particular among adolescents and adults), the necessity to monitor the impact of vaccine policy changes on disease incidence and the potential shift of morbidity towards older age groups, a national continuous surveillance of pertussis in general practice in mainland France was set up on 1 January 2017. This surveillance is conducted by the French Sentinelles network (réseau Sentinelles), a network of general practitioners (GPs). Here, we report analyses of the first 4 years of surveillance.

## Methods

### French Sentinelles network

The French Sentinelles network is a nationwide network of GPs taking part in epidemiological studies and in the continuous surveillance of various health indicators (10 in 2020: acute diarrhoea, acute respiratory infection, varicella, herpes zoster, Lyme disease, mumps, pertussis, sexually transmitted infections, suicidal attempts, influenza-like illness). GPs participate on a voluntary basis and report data (number of cases and description of cases) weekly via a secure Internet connection [13].

GPs in the Sentinelles network statistically differ from GPs in the country, in terms of their geographical distribution and number of consultations (slightly higher mean number of consultations per week for Sentinelles GPs, mainly due to consultations with patients under 14 years old). Due to this lack of representativeness, estimated incidence rates obtained by the network are partly corrected for bias by estimating the weighted incidence using external information for the GP population per region each year [14].

Pertussis surveillance in the network has been conducted since 2017. Each week, GPs report the number of confirmed pertussis cases they have observed (during office visits or home visits) and describe individual patients’ characteristics.

### Case definition

Pertussis cases are defined as patients for whom the GP suspects pertussis (there is no clinical definition specified, the suspicion is based on the clinician’s judgement) and who are laboratory confirmed or epidemiologically confirmed. Laboratory-confirmed cases are suspected pertussis cases confirmed with a positive PCR or a positive culture for *Bordetella*. Epidemiologically-confirmed cases are suspected pertussis cases who had contact in the 3 weeks before the beginning of their cough with a laboratory-confirmed case (the potential source of infection of this epidemiologically-confirmed case), or who had contact in the communicable period of the disease with someone who later presented signs of pertussis and had a laboratory confirmation (secondary case of this epidemiologically-confirmed case).

### Data collected

For each case, the following information is collected by GPs: age; sex (collected as a binary variable); presence of fever (no threshold temperature is specified to GPs participating in the surveillance); presence of cough, and, if present, characteristics of the cough and time between the beginning of the cough and the consultation (since 2019 only); hospitalisation requested by GP; microbiological examination (PCR or culture) and, if performed, the results, the name of the species identified and the time between the beginning of the cough and the sampling; presence of a relative or relatives with a history of cough. GPs also report if the patient has been vaccinated (‘yes’ if the patient has received at least one injection of pertussis vaccine), and if so briefly describe the immunisation schedule: type of last vaccine injected, date of last injection, number of dose(s), data collection method (i.e. vaccination data self-reported, based on the health booklet record, on the computerised medical record, or unknown). Surveillance questionnaires are reviewed every year and updated when needed.

### Data analysis

Data on pertussis cases reported to the Sentinelles network from 1 January 2017 to 31 December 2020 were used for the current analysis. Because of the coronavirus disease (COVID-19) pandemic, 2020 was separated from the period 2017–2019 for the study of monthly incidence rates.

Estimated weekly regional pertussis incidences were calculated as follows: the average number of cases notified by GPs for a given week and a given region (adjusted for participation) multiplied by the total number of GPs practicing in this region. In the same way, the monthly pertussis incidence corresponds to the average number of cases for a given month. Monthly cumulated pertussis incidence rates 2017–2019 per 100,000 inhabitants are the sum for each month of monthly incidences per 100,000 inhabitants for the considered month, over the period 2017–2019. National incidence rates are the sum of weekly and regional incidences calculated for a given year, divided by the total French population [14,15]. Confidence intervals (CI) were estimated under the assumption that the number of reported cases followed a Poisson distribution.

The characteristics of cases were described by raw numbers and percentages for qualitative variables, for the entire period and per year. Missing data (md) in the description of cases are specified in the ‘Results’ section and were not included in percentage calculations. Ages were described through minimum, maximum, median and interquartile range (IQR), and were divided in seven age groups for incidence calculation (0–11 months, 1–6 years, 7–13 years, 14–25 years, 26–45 years, 46–65 years, ≥ 66 years), based on the current French immunisation schedule.

Incidences were considered significantly different if their 95% confidence intervals (CI) did not overlap. Case distributions according to sex were tested through exact binomial tests.

All analyses were performed using R software version 3.6.0 [16].

## Results

### Incidences

In 2017, 458 GPs participated at least once in the surveillance, which represents a mean of 257 full-time equivalent GPs each week. These figures were respectively 480 and 259 in 2018, 552 and 295 in 2019, 685 and 347 in 2020. A total of 332 GPs participated throughout the whole period.

Between 2017 and 2020, French GPs reported a total of 132 confirmed pertussis cases (including 109 laboratory- and 23 epidemiologically-confirmed). National incidence rates per 100,000 inhabitants were estimated at 17 cases (95% CI: 12–22) in 2017, 10 (95% CI: 6–14) in 2018, 15 (95% CI: 10–20) in 2019 and 3 (95% CI: 1–5) in 2020, which translates into incidences of 11,238 cases (95% CI: 7,681–14,795) in 2017, 6,276 (95% CI: 3,585–8,967) in 2018, 10,130 (95% CI: 6,956–13,304) in 2019 and 1,910 (95% CI: 732–3,088) in 2020. Incidence rates of the first 3 years were not statistically different, but the 2020 incidence rate was significantly lower (Table 1). The highest incidence rate was observed in the 0 to 11 month-olds in 2017 (84; 95% CI: 0–204). The age group of 66 years old and older was the only age group with incidence rates lower than 10 per 100,000 each year.

**Table 1:** Number of cases and estimated incidence rates per 100,000 inhabitants of confirmed pertussis cases per age group, France, 2017–2020 (n = 132)

Age groups	2017		2018		2019		2020	
	N	Incidence rate per 100,000 inh. (95% CI)	N	Incidence rate per 100,000 inh. (95% CI)	N	Incidence rate per 100,000 inh. (95% CI)	N	Incidence rate per 100,000 inh. (95% CI)
0–11 months	2	84 (0–204)	0	0 (0–0)	0	0 (0–0)	1	23 (0–71)
1–6 years	10	40 (12–68)	3	11 (0–27)	6	32 (4–60)	2	8 (0–19)
7–13 years	7	28 (5–51)	3	17 (0–39)	4	14 (0–29)	2	3 (0–8)
14–25 years	6	16 (2–30)	5	11 (0–22)	7	19 (4–34)	1	2 (0–6)
26–45 years	10	17 (6–28)	10	18 (6–30)	11	15 (6–24)	0	0 (0–0)
46–65 years	8	12 (3–21)	5	4 (0–8)	17	22 (11–33)	3	3 (0–7)
≥ 66 years	3	8 (0–17)	3	3 (0–7)	1	1 (0–3)	2	4 (0–9)
**Total**	**46**	**17 (12**–**22)**	**29**	**10 (6**–**14)**	**46**	**15 (10**–**20)**	**11**	**3 (1**–**5)**

CI: confidence interval; Inh.: inhabitants; N: number of cases.

Over 2017–2019, the highest cumulated monthly incidence rates per 100,000 inhabitants were observed in June (8; 95% CI: 4–12), followed by May (5; 95% CI: 2–8) and July (4; 95% CI: 2–7). The lowest rates were observed in February (1; 95% CI: 0–3) and December (2; 95% CI: 0–4). The cumulated monthly incidence rate observed in June was significantly higher than the ones of February and December (Figure).

**Figure:**
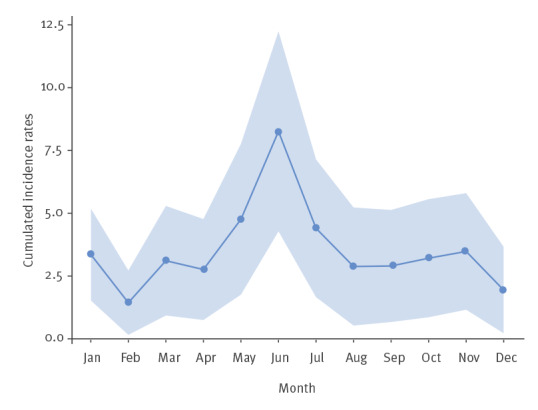
Monthly cumulated pertussis incidence rates per 100,000 inhabitants, France, 2017–2019^a^ (n = 121)

^a^ Because of the coronavirus disease (COVID-19) pandemic, the year 2020 was removed from the study period, for the analysis of monthly incidence rates of pertussis.

### Reported cases

There were no missing data on age and sex. Missing data on other variables are specified in the footnote of Table 2 and in Table 3. Women were significantly more represented among cases (63%; p= 0.004) than men. Most of cases were 15 years old or over (87/132; 66%). All cases had cough, with most having predominantly nocturnal cough (101/128; 79%), and cough attacks (122/132; 92%). Few of them (27/126; 21%) presented whooping cough, with a median age of 29 years old (range: 3 months–72 years old; IQR: 37) (Table 2 for cumulated 2017–2020 results, Supplementary Table S1 for results per year).

**Table 2:** Description of pertussis cases, France, 2017–2020 (n = 132)

Characteristics		Number (n = 132)	%
**Type of confirmation**			
Laboratory-confirmed case		109	83
Epidemiologically-confirmed case		23	17
**Demographic parameters**			
Median age (min–max; IQR)		31.5 years (2 months–87 years; 39 years)	
Sex	Women	83	63
	Men	49	37
**Clinical description**			
Presence of fever (as assessed by GP)		34	26
Hospitalisation		4	3
Cough		132	100
Predominantly nocturnal		101**^a^**	79
Cough with cough attacks		122**^a^**	92
Cough with frequent post-cough vomiting		39**^a^**	30
Cough leading to difficult breathing		54**^a^**	41
Cough with cyanosis		5**^a^**	4
Whooping cough		27**^a^**	21
Apnoea		15	12
**Immunisation status**			
Not vaccinated		54	48
Vaccinated		58	52
Unknown number of doses		21	36^b^
Known number of doses		37	64^b^
1 dose		1	3^c^
2 doses		1	3^c^
3 doses		8	22^c^
4 doses		13	35^c^
> 4 doses		14	38^c^
**Laboratory criteria**			
Culture prescribed		**6**	**5**
Culture performed		6	100^d^
Culture positive		6	100^e^
PCR prescribed		112	85
PCR performed		110	99^f^
PCR positive		103	94^g^

GP: general practitioner; IQR: interquartile range; max: maximum; min: minimum.

Missing data: Three (2%) for fever, four (3%) for ‘Predominantly nocturnal cough‘, two (2%) for ‘Cough with frequent post-cough vomiting‘, one (1%) for ‘Cough leading to difficult breathing‘, eight (6%) for ‘Cough with cyanosis‘, six (5%) for ‘Whooping cough‘, eight (6%) for ‘Cough with apnoea‘, four (3%) for hospitalisation, 20 (15%) for immunisation status, 21 of 58 (36%) for number of vaccine doses, one (1%) for PCR prescription, one of 112 (1%) for PCR performed, five (4%) for culture prescription.

^a^ Several characteristics can describe a same case, so the sum of characteristics exceeds the total number of cases.

^b^ The percentage is based on the total number of cases who were vaccinated (n = 58).

^c^ The percentage is based on the total number of cases with known number of vaccine doses received (n = 37).

^d^ The percentage is based on the total number of cases for whom culture was prescribed (n = 6).

^e^ The percentage is based on the total number of cases for whom culture was performed (n = 6).

^f^ The percentage is based on the total number of cases for whom PCR was prescribed (n = 112).

^g^ The percentage is based on the total number of cases for whom PCR was performed (n = 110).

**Table 3:** Details on vaccinated pertussis cases, per age group, France, 2017–2020 (n = 132)

Age group	Number of recommended injections (N_i_)^a^	Number of cases	Vaccinated cases with:					
			≥ 1 injections		≥ N_i_ injections	the last injection at the right age	≥ N_i_ injections and right date of last injection	last injection < 5 years ago
			Number (md)	%^b^	Number (md)	Number (md)	Number (md)	Number (md)
0–2 months	0	1	0 (0)	0	0 (0)	0 (0)	0 (0)	0 (0)
3–4 months	1	2	1 (0)	50	1 (0)	1 (0)	1 (0)	1 (0)
5–11 months	2	0	0 (0)	0	0 (0)	0 (0)	0 (0)	0 (0)
1–6 years	3	21	17 (0)	81	14 (3)	13 (4)	13 (4)	12 (4)
7–13 years	4	16	12 (1)	80	6 (5)	4 (6)	3 (7)	4 (6)
14–25 years	5	19	14 (3)	87	8 (5)	9 (4)	7 (5)	3 (4)
≥ 26 years	≥ 6	73	14 (16)	25	4 (8)	9 (1)	3 (8)	3 (1)
**Total**	NA	**132**	**58 (20)**	**52**	**33 (21)**	**36 (15)**	**27 (24)**	**23 (15)**

Md: number of vaccinated cases with missing data; NA: not applicable;

^a^ Based on the current pertussis immunisation schedule, presented in Box.

^b^ Percentages were calculated over the confirmed cases for whom vaccination status is known.

In 2019 and 2020, most of the cases (65%; 36/55) consulted a GP during the first 2 weeks after the beginning of their cough, 22% (12/55) in the first 7 days; 44% (24/55) between 7 and 14 days, 18% (10/55) waited 2 to 3 weeks and 16% (9/55) more than 3 weeks. This information was not collected in 2017 and 2018.

### Laboratory results

Most of the cases were confirmed by PCR (103/132; 78%). Among the 110 PCRs conducted, 103 (94%) were positive. Overall, 94% (83/88) of PCRs performed within the first 3 weeks from the beginning of the cough were positive, as well as 13 of 15 PCRs performed from samples collected more than 3 weeks after the beginning of the cough (for seven PCR performed, the time at which the cough had started before undertaking the PCR was missing). GPs gave little information on the results and the species targeted by the PCRs (details collected for only 34/110 PCR performed: 32 positive for *B. pertussis*, 2 positive for *B. parapertussis*). Among the 132 cases, only six cultures were prescribed (3 on vaccinated cases, 3 on unvaccinated cases). They were all positive, with three of six samplings conducted more than 2 weeks after the beginning of the cough (age of these cases: 11, 51 and 80 years old).

### Vaccination status

Approximately half of the cases (58/112; 52%) received at least one injection of pertussis vaccine. Most of vaccinated cases with available information (33/37) had received the number of doses recommended for their age by the current immunisation schedule. For the cases with data on the date of last injection (43 cases), the median duration between the last injection and the declaration of the case was 4.8 years (range: 0.2–31.1 years; IQR: 3.85). The vast majority of unvaccinated cases were 18 years old or older (45/54). Table 3 displays details on vaccinated cases, according to age group.

## Discussion

Since 1 January 2017, between 458 and 685 GPs per year have monitored pertussis in France. Results from the first 4 years of this surveillance indicate that over the 2017–2019 period, yearly incidence rates varied between 10 (95% CI: 6–14) and 17 (95% CI: 12–22) cases per 100,000 inhabitants, subsequently dropping to three cases per 100,000 inhabitants (95% CI: 1–5) in 2020. Cases between 2017 and 2020 were mainly women (63%), people aged 15 years or over (66%), and vaccinated people (52% of cases, among whom 33 of 37 with available information had received the number of doses recommended by the current immunisation schedule).

During this period, incidence data for pertussis in France were scarce. In 2013–2014, a study involving GPs estimated the pertussis French incidence rate to be 187.1 per 100,000 inhabitants (95% CI: 126.2–267.1) for people aged 50 years or over [9]. Our estimated annual incidence rates for people aged 46 years or over ranged between one (95% CI: 0–3) for ≥ 66 year-olds in 2019 and 22 (95% CI: 11–33) for 46–65 year-olds in 2019, which is significantly lower than this previous estimate. 

There could be several explanations for this difference. The first might be the cyclic nature of pertussis, where peaks in the numbers of cases occur every 3 to 5 years, with varying heights [17]. In France, a study based on laboratory data from people aged 2–20 years found that the circulation of *Bordetella* species was higher in 2012–2013 and in 2017–2019 than in the intermediate years [18]. According to data from the National Reference Center for Whooping Cough [19], the peak in 2012–2013 (154 and 153 *Bordetella pertussis* isolates for each respective year) might have been higher than a potential peak in 2017–2018 (65 and 64 *pertussis* isolates). Hence, the higher incidence rate reported in the 2013-2014 study [9] compared to our 2017–2019 incidence rates, might be due to the occurrence of natural fluctuations. In 2019 the number of isolates received by the laboratory appeared to slightly decrease (48 isolates). It should be noted that our study did not find significantly different incidence rates in 2017 (46; 95% CI: 12–22), 2018 (20; 95% CI: 6–14) and 2019 (15; 95% CI: 10–20). The size of our CIs did not allow detecting any trends between the 3 years. Another possible explanation for the higher pertussis incidence found in the 2013–2014 study, would be that in this first study, coughing patients were actively tested (an approach that generally leads to higher estimates than passive surveillance systems). The lower incidences reported from the present work could also reflect the impact of changes in vaccination policy, the improvement of vaccine coverage [11], or a lack of sensitivity or power of our surveillance system. Indeed, a report from 2021 based on serological analyses showed a 5.6% seropositivity rate for French adults aged 40–59 years old in 2015–16. This result suggests a high circulation of *Bordetella* in France (it may reflect the epidemic peak observed in the country in 2012–2013), tempered by the fact that analyses could not distinguish between a recent pertussis vaccination and a pertussis infection within the past 2 years [20].

It is highly probable that Sentinelles surveillance system, as well as the other pertussis surveillance systems running in France, underestimate the circulation of *Bordetella*, particularly among asymptomatic patients or patients presenting with atypical clinical symptoms. However, in another study published in 2021, the number of positive *B. pertussis* PCR tests obtained by two French laboratories (which carry out more than 90% of the testing for pertussis outside hospitals, in mainland France) were described between 2013 and 2020. In 2017, 2018, 2019 and 2020, respectively 3,354, 3,233, 2,783 and 606 positive PCRs were found [21]. Even taking into account that our surveillance system also includes cases confirmed by culture, as well as epidemiologically-confirmed cases, and recognising the large CIs, the incidences we found (11,238; 95% CI: 7,681–14,795 in 2017; 6,276; 95% CI: 3,585–8,967 in 2018; 10,130; 95% CI: 6,956–13,304 in 2019; and 1,910; 95% CI: 732–3,088 in 2020) are higher than incidences suggested by these laboratory results. This finding can suggest that Sentinelles GPs prescribe more pertussis PCRs than French GPs, thus better diagnosing this disease than French GPs. In Switzerland, where pertussis is, as in France, no longer a reportable disease, a comparable surveillance system is monitoring the disease since 1991, thanks to ca 200 voluntary GPs (Swiss Sentinel Surveillance Network – Sentinella). The last published annual incidence rate, 40 per 100,000 inhabitants in 2006 is of the same order of magnitude than what we observed [22]. In Europe, notification rates estimated by the ECDC were 9.4 in 2017, and 7.9 in 2018 [23], also of the same order of magnitude than our findings. Individuals aged 15 years old or over accounted for 62% of all European cases reported in 2017 and 2018, a figure comparable with our result of 66% of cases aged 15 years old or over, indicating a shift in morbidity towards older age groups, already observed in several countries [24-26]. 

According to the PERTINENT surveillance system, a significant decrease in pertussis incidence rates was observed in France in infants between 2017 (49.1; 95% CI: 39.5–60.3) and 2018 (34.7; 95% CI: 26.7–44.4), corresponding to an incidence rate ratio of 0.71 (p = 0.034) [7]. Between these 2 years, incidence rate ratios were 1.43 in Czechia (p = 0.468), 0.25 in Catalonia, Spain (p = 0.002), 0.21 in Navarra, Spain (p = 0.148), 0.14 in Ireland (p = 0.002), 0.63 in Italy (p = 0.053), and zero in Norway [7]. Our estimates of incidence for infants in 2017 (84; 95% CI: 0–204) were comparable to those of PERTINENT estimates, while they were well below for 2018 (0; 95% CI: 0–0). As PERTINENT, we observed a significant decreasing trend between incidences in infants in 2017 and 2018, which could be linked with the natural cycle of pertussis. In France, some infants are followed by paediatricians in private practices instead of GPs (mainly in large cities), and the hospitalisation of all pertussis cases under 1 year old is recommended. We can therefore hypothesise that for this population, a surveillance system in general practice leads to an underestimation of the incidence in the community. A surveillance system in hospitals might be more appropriate.

We found a 2020 incidence rate significantly lower than the ones of the 3 previous years, with only 11 declared cases (no cases declared from mid-April to December 2020). As in most of the countries, drastic physical distancing and hygiene measures were set up in France from March 2020, because of the COVID-19 pandemic. Several published studies already showed the reduction of the circulation of other respiratory pathogens in 2020, such as influenza viruses and respiratory syncytial virus in Australia [27,28] and Finland [29]. It is highly probable that all the measures set up to address COVID-19 contributed to lower the circulation of pertussis in 2020, but we can also hypothesise that the heightened focus on respiratory viruses in that year, particularly on severe acute respiratory syndrome coronavirus 2 (SARS-CoV-2), may have interfered with pertussis diagnosis efforts.

We found a tendency to higher incidence rates between May and July. Several recently published studies found such a summer-seasonal pattern in America [30], Europe [31,32], Asia [33] or Oceania [24]. This seasonality is still not well explained. Some experts hypothesise that there might be an under-reporting during winter months, when clinical presentation can be misdiagnosed with circulating winter respiratory viruses [32]. Climate or environmental factors could also be at play, as well as changes in host behaviours [30].

Our results also showed that women were significantly more numerous than men among cases (p = 0.004). This finding is consistent with a meta-analysis realised in nine countries, which found an excess pertussis incidence rate in females for all investigated age groups, particularly infants and very young children. This was unlikely to be due to differences in exposure, but the underlying mechanism is still unknown. Possible explanations include behavioural factors, which may contribute to some of the differences seen in the post-pubertal age groups, genetic factors, as well as sex hormones [25].

We found a 52% vaccination rate among cases, with most of them having received the right number of doses, and the last dose at the recommended age, which may reflect fast waning of immunity after vaccination. Half of the vaccinated cases had received their last injection less than 4.8 years before declaration. A study published in 2022 showed a fast decay of vaccine protection among children having followed the current French immunisation schedule [18]. The data collected in the current study (number of injections, date of last injection), however, do not include the date of all injections, and therefore do not allow to know if the full recommended immunisation schedule was adhered to. Moreover, the identified *Bordetella* species is specified for a minority of our cases. Some of these vaccinated cases might therefore have been infected by *B. parapertussis* or *B. holmesii*, which may partly explain our findings. For unvaccinated cases, we have no information on the level of reliability of the information collected, and we do not know whether the GP had access to paper or electronic health records for their patient, or only relied on the declaration of the patient, with possible recall bias, which could lead to an underestimation of the percentage of cases having received at least one injection of vaccine, especially if the last injection occurred in early childhood. It is plausible that a number of cases reported as ‘unvaccinated’ in our study did not receive a booster at adult age, but were vaccinated in early childhood.

As previously stated, the Sentinelles surveillance system presents limitations, which lead to a possible underestimation of the pertussis circulation in France. We can mention for instance the under-prescription of pertussis PCR or culture analysis by GPs for coughing patients, and the fact that many people (specially adolescents and adults) may not consult GPs, because of mild symptoms. The Sentinelles surveillance system is moreover aligned on recommendations made by French authorities, and relies on the confirmation of cases by PCR or culture, which is sensitive only for cases with recent cough. French authorities decided to dissuade GPs from using serological analyses for pertussis diagnosis, and to stop reimbursement by national health insurance of these analyses in March 2011, because of the lack of sensitivity and specificity of commercial tests available at this time, and of difficulties in the interpretation of results for patients recently vaccinated [34]. As a consequence, serological analyses are not frequently currently used in France, and were not included in the case definition of our surveillance. We can nevertheless notice that serological analyses are commonly used for diagnosis and surveillance in other countries, and are included in pertussis case definitions for ECDC and World Health Organization surveillance [23,35]. Many Sentinelles GPs reported that they often do not prescribe PCR analyses of suspected coughing cases, because they had already been coughing for more than 3 weeks. The cause of this may be that in France, PCR analyses are not reimbursed by the national health insurance if the cough has lasted for more than 3 weeks. We nevertheless observed a high PCR positivity rate (13/15) among cases sampled after 3 weeks of cough. However, this result might have been inflated by the fact that only laboratory-confirmed or epidemiologically-confirmed cases are targeted by our surveillance system (which implies that negative results are more unlikely to be declared). Unfortunately, we do not know the targets of PCR used for testing cases, and rarely know which *Bordetella* species was identified. The cases with a positive PCR after 3 weeks of cough could have been infected by *B. holmesii*, which is known to stay longer in the respiratory tract [36]. In France, PCR analyses are not reimbursed by the national health insurance for cases vaccinated since less than 3 years, which may lead to an under-detection of cases among recently vaccinated people. The vaccination rate we observed among our cases might therefore be underestimated in comparison with the real situation in the community. The culture is reimbursed, but few laboratories undertake this analysis, because of its cost and complexity. Some GPs still prescribe serological analyses, but positive results are not included in our surveillance system. Prescription habits have evolved since the Sentinelles surveillance started, and GPs have progressively been incited to prescribe PCR or culture analyses to people with a recent cough and who are related to suspected cases with a long-lasting cough. While this prescription-behaviour change over time may lead to an improvement of pertussis diagnosis, particularly among adults, it may give the false impression that the disease incidence is increasing in the adult population. We can also notice that because of a limited number of participating GPs, CIs of estimated incidence rates that we obtained are wide. Because of a small number of pertussis cases declared, as well as the only partially-corrected bias due the non-representativeness of Sentinelles GPs, and the presence of missing data (particularly on vaccination data), the results presented here must be interpreted with caution.

Even if non exhaustive, the observations of this surveillance system are of great interest to describe the evolution of the number and characteristics of cases (particularly clinical data and vaccination status), and to follow the evolution of the distribution of cases among age groups.

## Conclusions

Our results indicate that current incidences of pertussis cases observed in general practice are low in France. Cases are mainly adults, and a high vaccination rate was observed among cases. These findings can be partly explained by the fact that some children (particularly in big cities) visit paediatricians in France rather than GPs. They also reflect the high vaccination coverage among children and may suggest a fast waning of immunity after vaccination. As the immunisation policy recently evolved and the coverage rates are improving among children and adults, the continuation of this surveillance will be relevant to assess in a few years the impact of these changes on the disease incidence.

## Supplementary Data

90000 Supplementary Materials

## References

[1] Bonmarin I Bouraoui L Guiso N Levy-Bruhl D . La coqueluche: collecte de données et choix des stratégies vaccinales [Pertussis: data collection and vaccinal strategy]. Med Mal Infect. 2009;39(5):271-7. 10.1016/j.medmal.2009.02.009 19362438

[2] Guiso N de La Rocque F Njamkepo E Lécuyer A Levy C Romain O French Pediatrics Groups Association Clinique et Thérapeutique Infantile du Val de Marne and Association Française de Pédiatrie Ambulatoire . Pertussis surveillance in private pediatric practices, France, 2002-2006. Emerg Infect Dis. 2008;14(7):1159-61. 10.3201/eid1407.071246 18598649 PMC2600362

[3] Aguas R Gonçalves G Gomes MG . Pertussis: increasing disease as a consequence of reducing transmission. Lancet Infect Dis. 2006;6(2):112-7. 10.1016/S1473-3099(06)70384-X 16439331

[4] Karsenty J-Y, Roure C, Vidal-Trecan G. La coqueluche en France. [Whooping cough in France]. BEH. 1990;19.

[5] Bégué P, Grimpel E, Roure C, Guiso N. La coqueluche en France: Nécessité de mise en place d'une surveillance. [Whooping cough in France: need for establishing a surveillance system]. BEH. 1992;48.

[6] Tubiana S Belchior E Guillot S Guiso N Lévy-Bruhl D Renacoq Participants . Monitoring the Impact of Vaccination on Pertussis in Infants Using an Active Hospital-based Pediatric Surveillance Network: Results from 17 Years’ Experience, 1996-2012, France. Pediatr Infect Dis J. 2015;34(8):814-20. 10.1097/INF.0000000000000739 25955837

[7] Merdrignac L Aït El Belghiti F Pandolfi E Jané M Murphy J Fabiánová K PERTINENT Group PERTINENT group . Incidence and severity of pertussis hospitalisations in infants aged less than 1 year in 37 hospitals of six EU/EEA countries, results of PERTINENT sentinel pilot surveillance system, December 2015 to December 2018. Euro Surveill. 2021;26(4). 10.2807/1560-7917.ES.2021.26.4.1900762 33509338 PMC7848786

[8] Guiso N Levy C Romain O Guillot S Werner A Rondeau MC Whooping cough surveillance in France in pediatric private practice in 2006-2015. Vaccine. 2017;35(45):6083-8. 10.1016/j.vaccine.2017.09.072 28974408

[9] Guiso N Gallais JL Gavazzi G Pinquier D Gaillat J . Incidence of pertussis in subjects aged 50years and older in France in 2013-2014. Med Mal Infect. 2018;48(1):30-6. 10.1016/j.medmal.2017.09.002 29037454

[10] Santé publique France (SPF). Couverture vaccinale [Vaccination rate]. Bulletin de Santé Publique Edition Nationale. 2021. Available from: https://professionnels.vaccination-info-service.fr/var/vis/storage/original/application/download/BSP_nat_vaccination_180521.pdf

[11] Cohen R Gaudelus J Denis F Stahl JP Chevaillier O Pujol P Pertussis vaccination coverage among French parents of infants after 10years of cocoon strategy. Med Mal Infect. 2016;46(4):188-93. 10.1016/j.medmal.2016.03.005 27132209

[12] Marchal C Belhassen M Guiso N Jacoud F Van Ganse E Le Pannerer M Vaccination coverage rates for Diphtheria, Tetanus, Poliomyelitis and Pertussis booster vaccination in France between 2013 and 2017: Learnings from an analysis of National Health System Real-World Data. Vaccine. 2021;39(3):505-11. 10.1016/j.vaccine.2020.12.021 33357956

[13] Flahault A Blanchon T Dorléans Y Toubiana L Vibert JF Valleron AJ . Virtual surveillance of communicable diseases: a 20-year experience in France. Stat Methods Med Res. 2006;15(5):413-21. 10.1177/0962280206071639 17089946

[14] Souty C Turbelin C Blanchon T Hanslik T Le Strat Y Boëlle PY . Improving disease incidence estimates in primary care surveillance systems. Popul Health Metr. 2014;12(1):19. 10.1186/s12963-014-0019-8 25435814 PMC4244096

[15] Institut national de la statistique et des études économiques (INSEE). Estimation de la population au 1ᵉʳ janvier 2021 - Séries par région, département, sexe et âge de 1975 à 2021. [Population estimate on 1 January 2021, results by region, department, sex and age from 1975 to 2021]. French. [accessed 30 Jan 2021]. Available from: https://www.insee.fr/fr/statistiques/1893198#consulter.

[16] R Core Team. (2020). R: A language and environment for statistical computing. R Foundation for Statistical Computing, Vienna, Austria

[17] Tan T Dalby T Forsyth K Halperin SA Heininger U Hozbor D Pertussis Across the Globe: Recent Epidemiologic Trends From 2000 to 2013. Pediatr Infect Dis J. 2015;34(9):e222-32. 10.1097/INF.0000000000000795 26376316

[18] Paireau J Guillot S Aït El Belghiti F Matczak S Trombert-Paolantoni S Jacomo V Effect of change in vaccine schedule on pertussis epidemiology in France: a modelling and serological study. Lancet Infect Dis. 2022;22(2):265-73. 10.1016/S1473-3099(21)00267-X 34672963

[19] National Reference Center for Whooping Cough and other Bordetella Infections. 2019 annual summary report. Paris: Institut Pasteur; 2020. Available from: https://www.pasteur.fr/fr/sante-publique/CNR/les-cnr/coqueluche-et-autres-bordetelloses/rapports-d-activite.

[20] Berbers G van Gageldonk P Kassteele JV Wiedermann U Desombere I Dalby T Serosurveillance Study Team . Circulation of pertussis and poor protection against diphtheria among middle-aged adults in 18 European countries. Nat Commun. 2021;12(1):2871. 10.1038/s41467-021-23114-y 34001895 PMC8128873

[21] Matczak S Levy C Fortas C Cohen JF Béchet S El Belghiti FA Association between the COVID-19 pandemic and pertussis in France using multiple nationwide data sources. Preprint. 2021. Available at: https://www.medrxiv.org/content/10.1101/2021.07.16.21260367v1.full.pdf 10.1101/2021.07.16.21260367 PMC9229195 35748301

[22] Wymann MN Richard J-L Vidondo B Heininger U . Prospective pertussis surveillance in Switzerland, 1991-2006. Vaccine. 2011;29(11):2058-65. 10.1016/j.vaccine.2011.01.017 21251904

[23] European Centre for Disease Prevention and Control (ECDC). Pertussis - Annual Epidemiological Report for 2018. Stockholm: ECDC; 2020. Available from: https://www.ecdc.europa.eu/sites/default/files/documents/AER_for_2018_pertussis.pdf. 2020.

[24] Leong RNF Wood JG Turner RM Newall AT . Estimating seasonal variation in Australian pertussis notifications from 1991 to 2016: evidence of spring to summer peaks. Epidemiol Infect. 2019;147:e155. 10.1017/S0950268818003680 31063086 PMC6518527

[25] Peer V Schwartz N Green MS . A multi-country, multi-year, meta-analytic evaluation of the sex differences in age-specific pertussis incidence rates. PLoS One. 2020;15(4):e0231570. 10.1371/journal.pone.0231570 32324790 PMC7179848

[26] Skowronski DM De Serres G MacDonald D Wu W Shaw C Macnabb J The changing age and seasonal profile of pertussis in Canada. J Infect Dis. 2002;185(10):1448-53. 10.1086/340280 11992280

[27] Yeoh DK Foley DA Minney-Smith CA Martin AC Mace AO Sikazwe CT The impact of COVID-19 public health measures on detections of influenza and respiratory syncytial virus in children during the 2020 Australian winter. Clin Infect Dis. 2021;72(12):2199-202. 10.1093/cid/ciaa1475 32986804 PMC7543326

[28] Sullivan SG Carlson S Cheng AC Chilver MB Dwyer DE Irwin M Where has all the influenza gone? The impact of COVID-19 on the circulation of influenza and other respiratory viruses, Australia, March to September 2020. Euro Surveill. 2020;25(47). 10.2807/1560-7917.ES.2020.25.47.2001847 33243355 PMC7693168

[29] Kuitunen I Artama M Mäkelä L Backman K Heiskanen-Kosma T Renko M . Effect of Social Distancing Due to the COVID-19 Pandemic on the Incidence of Viral Respiratory Tract Infections in Children in Finland During Early 2020. Pediatr Infect Dis J. 2020;39(12):e423-7. 10.1097/INF.0000000000002845 32773660

[30] Bhatti MM Rucinski SL Schwab JJ Cole NC Gebrehiwot SA Patel R . Eight-Year Review of Bordetella pertussis Testing Reveals Seasonal Pattern in the United States. J Pediatric Infect Dis Soc. 2017;6(1):91-3. 26621328 10.1093/jpids/piv079

[31] Hitz DA Tewald F Eggers M . Seasonal Bordetella pertussis pattern in the period from 2008 to 2018 in Germany. BMC Infect Dis. 2020;20(1):474. 10.1186/s12879-020-05199-w 32620085 PMC7333396

[32] De Greeff SC Dekkers AL Teunis P Rahamat-Langendoen JC Mooi FR De Melker HE . Seasonal patterns in time series of pertussis. Epidemiol Infect. 2009;137(10):1388-95. 10.1017/S0950268809002489 19327200

[33] Zeng Q Li D Huang G Xia J Wang X Zhang Y Time series analysis of temporal trends in the pertussis incidence in Mainland China from 2005 to 2016. Sci Rep. 2016;6(1):32367. 10.1038/srep32367 27577101 PMC5006025

[34] Rossignol L Debin M Brisse S Guillot S Toubiana J Ait El Belghiti F La coqueluche : histoire d'une maladie infantile. [Pertussis: history of an infantile disease]. Exercer. 2019;150:82-8. French.

[35] World Health Organisation (WHO). Pertussis - Surveillance standards. Geneva: WHO. [Accessed 3 Apr 2022]. Available from: https://www.who.int/immunization/monitoring_surveillance/burden/vpd/WHO_SurveillanceVaccinePreventable_16_Pertussis_R1.pdf?ua=1

[36] Nguyen LB Epelboin L Gabarre J Lecso M Guillot S Bricaire F Recurrent Bordetella holmesii bacteremia and nasal carriage in a patient receiving rituximab. Emerg Infect Dis. 2013;19(10):1703-5. 10.3201/eid1910.130345 24050722 PMC3810743

[37] Haute Autorité de Santé. Recommandation vaccinale contre la coqueluche chez la femme enceinte. [Recommended immunisation schedule against pertussis for pregnant women]. 2022. French. Available from: https://www.has-sante.fr/upload/docs/application/pdf/2022-04/recommandation_vaccinale_contre_la_coqueluche_chez_la_femme_enceinte.pdf

