# Supplementary material for: Pertussis surveillance results from a French general practitioner network, France, 2017 to 2020
Source: Euro Surveill. 2022 Apr 28;27(17):2100515. doi: 10.2807/1560-7917.ES.2022.27.17.2100515 (PMC9052767; doi:10.2807/1560-7917.ES.2022.27.17.2100515)
Supplement: Supplementary Materials [file 2100515_SupplementaryMaterials.pdf]

Supplementary Table S1: Description of pertussis cases per year, from 2017 to 2020

This supplementary material is hosted by Eurosurveillance as supporting information alongside the article [Pertussis surveillance results from a French general practitioner network, France, 2017 to 2020], on behalf of the authors, who remain responsible for the accuracy and appropriateness of the content. The same standards for ethics, copyright, attributions and permissions as for the article apply. Supplements are not edited by Eurosurveillance and the journal is not responsible for the maintenance of any links or email addresses provided therein.

	2017		2018		2019		2020	
	**N**	**%**	**N**	**%**	**N**	**%**	**N**	**%**
**Total**	**46**		**29**		**46**		**11**	
Laboratory confirmed case	39	85	23	79	38	83	9	82
Epidemiological case	7	15	6	21	8	17	2	18
**Clinical description**								
Age : median in years (min – max in years, m for months; IQR in years)	17.5 (2m – 80; 35.75)		31 (4 – 75; 34)		37 (2 – 75; 37.5)		18 (3m – 87; 43)	
Sex (female)	29	63	20	69	28	61	6	55
Fever	18	39	7	25	6	14	3	27
Hospitalization	3	7	0	0	0	0	1	9
Cough	46	100	29	100	46	100	11	100
**Characteristics of the cough***								
Predominantly nocturnal	36	78	24	83	34	81	7	64
Cough with cough attacks	45	98	29	100	41	89	7	64
Cough with frequent post-cough vomiting	14	31	10	34	12	27	3	27
Cough leading to difficult breathing	19	41	12	41	20	44	3	27
Cough with cyanosis	1	2	1	4	1	2	2	18
Whooping cough	6	14	4	14	14	31	3	27
Apnea	7	16	4	14	3	7	1	10
**Immunization status**								
Not vaccinated	17	44	11	44	19	51	7	64
Vaccinated	22	56	14	56	18	49	4	36
**Characteristics of vaccinated cases**								
Unknown number of doses	7	32	4	29	7	39	3	75
Number of doses known	15	68	10	71	11	61	1	25
1 dose (n/number of doses known)	1	7	0	0	0	0	0	0
2 doses (n/number of doses known)	0	0	0	0	1	9	0	0
3 doses (n/number of doses known)	4	27	2	20	2	18	0	0
4 doses (n/number of doses known)	5	33	2	20	5	45	1	100
> 4 doses (n/number of doses known)	5	33	6	60	3	27	0	0
**Biology**								
Culture prescribed	3	7	1	4	1	2	1	9
Culture performed (n/prescribed)	3	100	1	100	1	100	1	100
Culture positive (n/performed)	3	100	1	100	1	100	1	100
PCR prescribed	40	87	25	86	38	84	9	82
PCR performed (n/prescribed)	39	100	24	96	38	100	9	100
PCR positive (n/performed)	36	92	22	92	37	97	8	89

Missing data for 2017: 1 (2%) for 'Cough with frequent post-cough vomiting', 4 (9%) for 'Cough with cyanosis', 4 (9%) for 'Whooping cough', 3 (7%) for 'Apnea', 7 (15%) for immunization status, 7 out of 22 (32%) for number of vaccine doses, 1 (2%) for PCR performed, 1 (2%) for culture prescription

Missing data for 2018: 1 (3%) for fever, 1 (3%) for hospitalization, 3 (10%) for 'Cough with cyanosis', 1 (3%) for 'Whooping cough', 1 (3%) for 'Apnea', 4 (14%) for immunization status, 4 out of 14 (29%) for number of vaccine doses, 1 (3%) for culture prescription

Missing data for 2019: 2 (4%) for fever, 3 (7%) for hospitalization, 4 (9%) for 'Predominantly nocturnal cough', 1 (2%) for 'Cough with frequent post-cough vomiting', 1 (2%) for 'Cough leading to difficult breathing', 1 (2%) for 'Cough with cyanosis', 1 (2%) for 'Whooping cough', 3 (7%) for 'Apnea', 9 (20%) for immunization status, 7 out of 18 (39%) for number of vaccine doses, 1 (2%) for PCR prescription, 3 (7%) for culture prescription

Missing data for 2020: 1 (9%) for 'Apnea', 3 out of 4 (75%) for number of vaccine doses

* Several characteristics can describe a same case, so the sum of characteristics exceeds the total number of cases
